# Protein-Protein Interactions at the Adrenergic Receptors

**DOI:** 10.2174/138945012798868489

**Published:** 2012-01

**Authors:** Susanna Cotecchia, Laura Stanasila, Dario Diviani

**Affiliations:** 1 Départment de Pharmacologie et de Toxicologie, Université de Lausanne, Switzerland; 2 Dipartimento di Fisiologia Generale e Ambientale, Università di Bari, Italy

**Keywords:** Adrenergic receptor subtypes, signaling complexes, arrestins, receptor oligomerization.

## Abstract

The adrenergic receptors are among the best characterized G protein-coupled receptors (GPCRs) and knowledge on this receptor family has provided several important paradigms about GPCR function and regulation. One of the most recent paradigms initially supported by studies on adrenergic receptors is that both βarrestins and G protein-coupled receptors themselves can act as scaffolds binding a variety of proteins and this can result in growing complexity of the receptor-mediated cellular effects. In this review we will briefly summarize the main features of βarrestin binding to the adrenergic receptor subtypes and we will review more in detail the main proteins found to selectively interact with distinct AR subtype. At the end, we will review the main findings on oligomerization of the AR subtypes.

## INTRODUCTION

The adrenergic receptors (AR) mediate the functional effects of catecholamines, like epinephrine and norepinephrine, by coupling to different signaling pathways modulated by G proteins. The adrenergic receptor family includes nine different gene products, three β (β_1_, β_2_, β_3_), three α_1_ (α_1a _, α_1b _and α_1d_) and three α_2_ (α_2A_, α_2B _and α_2C_) receptor subtypes.

The adrenergic receptors, and in particular the β_2_AR, are among the best characterized G protein-coupled receptors (GPCRs) and knowledge on this receptor family has provided several important paradigms about GPCR function and regulation.

One of the most recent paradigms initially supported by studies on adrenergic receptors is that G protein-coupled receptors can act as scaffolds binding a variety of proteins and this can promote multiple signaling events which results in growing complexity of the receptor-mediated cellular effects (reviewed in ref. [1]). 

In the late 1980s, the first protein found to interact with the β_2_AR, beyond G proteins, was βARK1 (βAR kinase 1) [2], discovered as the first member of the G protein-coupled receptor kinase family (GRK) [3]. The ability of GRK2 and 3 to selectively interact with the agonist-bound form of the β_2_AR was a crucial observation to identify their role in homologous desensitization. 

Soon after the discovery of GRKs, it became apparent that β_2_AR desensitization required also the interaction of an arrestin protein with the phoshorylated receptor [2, 4]. This interaction resulted in both receptor-G protein uncoupling and receptor endocytosis into clathrin-coated pits [5]. 

In the last ten years, an important function of βarrestins has come to light as scaffolds for a growing number of signaling proteins thus coordinating complex signal transduction events [6]. In particular, it is well established that βarrestins are scaffolds for components of the mitogen activated protein kinase (MAPK) cascade thus mediating MAPK activation induced by various GPCRs.

This seminal work encouraged the search for novel protein-protein interactions at several GPCRs with the aim of identifying previously unappreciated signaling mechanisms that might represent new targets of pharmacological intervention. A number of approaches have been followed to identify novel proteins interacting with the receptors, including yeast two-hybrid screen using cytosolic portions of the receptors as bait, pull-down or *in vitro* overlay assays using purified proteins, co-immunoprecipitation of receptor-protein complexes from recombinant or native cells, FRET (fluorescence resonance energy transfer) or BRET (bioluminescence resonance energy transfer) technology in cells. These studies resulted in the identification of a variety of proteins interacting with the adrenergic receptors, several of them in a receptor subtype selective pattern. A major challenge faced by these studies has been to identify the functional implications of these interactions. Some interacting proteins have been found to either promote or impair receptor-mediated signaling whereas others are involved in receptor trafficking or endocytosis. 

Among the protein-protein interactions found to regulate GPCR function, receptor oligomerization has been extensively investigated in recent years [7]. Both homo- and hetero-oligomerization have been reported for different adrenergic receptor subtypes using different experimental approaches. This phenomenon seems to have implications in different aspects of receptor function, including its pharmacological profile, signaling, trafficking or endocytosis.

The canonical interactions of the adrenergic receptors with G proteins, GRKs and βarrestins have been extensively studied and exhaustively reviewed elsewhere [8, 9]. In this review we will briefly summarize the main features of βarrestin binding to the adrenergic receptor subtypes and we will review, more in detail, a number of proteins found to selectively interact with distinct AR subtype. At the end, we will review the main findings on oligomerization of the AR subtypes. Considering the large number of studies on protein-protein interactions at GPCRs, our review might not systematically include all published data. 

The direct interaction of GPCRs with selected partners has recently emerged as a new mechanism of receptor signaling and regulation. Since these mechanisms might be specific for distinct receptors or cell types, the study of these interactions has interesting implications in pharmacology and drug development. 

## βARRESTIN INTERACTION WITH THE AR SUBTYPES

The well established crucial role played by βarrestin1 and 2 in coordinating a variety of signaling networks might imply that these proteins can form macromolecular complexes with virtually any GPCR. 

The interaction of βarrestins with the β_2_AR has been extensively characterized both at functional and molecular level using different approaches including *in vitro* binding of purified proteins, co-immunoprecipitation, BRET or FRET, βarrestin translocation as well as confocal microscopy to assess colocalization of the proteins [6, 8, 9]. The β_2_AR displays a pattern of interaction with βarrestins defined as "Class A" characterized by greater affinity for βarrestin 2 than 1 and a short-lived receptor/βarrestin complex leading to rapid receptor recycling after endocytosis. The interaction with βarrestins is important in mediating β_2_AR-induced activation of ERK1/2 (see references in [6]).

In contrast to the significant amount of data on the β_2_AR, much less is known about the interaction of βarrestins with other AR subtypes. The interaction of the β_1_AR with βarrestin is much weaker than that displayed by the β_2_AR subtype and this seems to correlate with the resistance of the β_2_AR to undergo agonist-induced endocytosis [10]. However, the β_1_AR can transactivate the epithelial growth factor (EGF) receptor in a βarrestin-dependent mechanism and this effect might have implications in cardioprotection [11]. This is suggested by a recent study reporting that recruitment of βarrestin to the C-tail of the β_1_AR is required for maintaining the β_1_AR/EGF receptor complex.

Also the β_3_AR does not seem to interact with βarrestins as suggested by two lines of evidence. First, the β_3_AR is resistant to agonist-induced endocytosis [12]. Second, the ability of the β_3_AR to activate MAPK is independent from βarrestin binding since its activation does not result in βarrestin recruitment to the plasma membrane [13].

The interaction of βarrestin with the α_2_AR was initially investigated measuring the effect of overexpressed βarrestin on receptor endocytosis [14]. Overxpression of βarrestin significantly increased the endocytosis of the α_2B_ and α_2C_AR, but had no effect on the α_2A_AR suggesting poor interaction of this receptor subtype with βarrestin. The lack of βarrestin interaction with the α_2A_AR was confirmed by *in vitro* studies measuring the binding of purified βarrestin to peptides derived from the 3i loop of the three α_2_AR subtypes [15]. 

The interaction of the α_1a_ and α_1b_AR subtypes with βarrestin has been investigated by a recent study using different approaches including co-immunoprecipitation, endocytosis and confocal microscopy [16]. Whereas the α_1b_AR displayed robust agonist-induced endocytosis, the α_1a_AR did not. The results from both co-immunoprecipitation experiments and βarrestin translocation assays indicated that the agonist-induced interaction of the α_1a_AR with βarrestin was much weaker than that of the α_1b_AR. The interaction of βarrestin with the α_1d_AR has not been directly explored so far.

Altogether these findings indicate that the interaction pattern of βarrestin with distinct AR subtypes is divergent and correlates with differences in the internalization properties of the receptors. Overall, despite the large number of studies on the β_2_AR, a lot remains to be explored concerning the implications of βarrestin in adrenergic receptor function and regulation.

## PROTEINS INTERACTING AT THE β_1_AR 

The first cytoplasmic proteins found to interact selectively with the β_2_AR subtype were endophilins 1/2/3, also called SH3p4/p8/p13 [17]. This SH3 domain-containing protein family bound the proline-rich third intracellular (i3) loop of the β_2_AR in both pull-down assays and yeast two hybrid screens. The primary effect of this interaction was to promote agonist-induced internalization of the receptor and to modestly decrease its coupling to Gs. Intriguingly, a similar proline-rich sequence is found in the i3 loop of the β_3_ or β_2A_AR, but these receptors do not bind endophilins. The mechanism through which endophilin might regulate receptor coupling and internalization is not known, but it could include steric hindrance or allosteric modulation of G protein and arrestin binding.

A second class of proteins selectively interacting with the β_1_AR are proteins containing the PDZ (PSD-95/Discslarge/ZO-1 homology) domain which recognizes the extreme C-terminus of the target protein. The β_1_AR possesses a type I PDZ binding sequence, E-S-K-V, at the end of its C-tail and it has been shown to bind the postsynaptic protein PSD-95 [18]. This protein is abundant in brain where it co-localizes with the β_1_AR in postsynaptic densities. This interaction was found in a yeast two-hybrid screening using the β_1_AR C-terminus as bait and it markedly attenuated β_1_ internalization, while having no impact on the receptor desensitization or signaling to adenylate cyclase. Association with PSD-95 could also facilitate the linkage of the β_1_ to NMDA glutamate receptors, known to be regulated by adrenergic signaling in the brain, and, more in general, to facilitate targeting of the β_1_AR subtype at synapses. Other studies demonstrated that the interaction with PSD-95 is negatively regulated by agonist treatment, through phosphorylation of the receptor by GRK5, thus allowing receptor internalization [19]. 

A second PDZ domain containing protein interacting with the β_1_AR is membrane-associated guanylate kinase inverted-2 (MAGI-2), a multidomain scaffold protein, also known as S-SCAM (synaptic scaffolding molecule). The first PDZ domain of MAGI-2 binds with high affinity to the β_1_AR C-tail, as demonstrated by overlay and pull-down techniques [20]. The β_1_AR/MAGI-2 interaction occurred constitutively in cells, but it was further enhanced by isoproterenol treatment. It favored agonist-induced internalization of the receptor, an effect opposite to the one observed for PSD-95, another PDZ-domain protein [18]. MAGI-2 also promoted β_1_AR association to βcatenin, a known MAGI-2 partner. 

A third PDZ domain containing protein binding the β_1_AR is GIPC (Gα-interacting protein-interacting protein, C terminus) [21]. This interaction decreased ERK1/2 activity stimulated by the β_1_AR, through a Gi-dependent process, with no observable effects on the Gs-mediated cAMP accumulation or on receptor internalization. One last example of a PDZ domain-mediated interaction with the β_1_AR is CAL (Cystic Fibrosis Transmembrane Conductance Regulator-Associated Ligand), found by pull-down techniques to interact with the ESKV sequence of the β_1_AR [22]. Over-expression of CAL, a protein localized in the Golgi apparatus, reduced surface expression of the receptor, a process competitively reversed by PSD-95.

GPCRs can indirectly activate Ras through the Gβγ subunits of the G protein that can recruit c-Src, Grb-Sos and PI3 kinase. The β_1_AR was the first GPCR for which a direct interaction with a Ras-GEF (GTP exchange factor), CNrasGEF, had been described [23]. CNrasGEF bound the ESKV sequence of the β_1_AR through its PDZ domain and mediated isoproterenol-induced Ras activation. 

The importance of the PDZ binding sequence in the C-tail of the β_1_AR was highlighted by findings obtained expressing the receptor in mouse cardiac myocytes [24]. In these cells, stimulation of the β_1_AR increases the contraction rate, whereas the β_2_AR has a biphasic effect with an initial increase followed by a decrease mediated by receptor coupling to Gi. In addition, whereas the β_2_AR undergoes endocytosis, the β_1_AR does not. Interestingly, the mutation of the PDZ binding sequence of the β_1_AR enabled both receptor internalization and coupling to Gi thus providing evidence that differences in the interaction with PDZ containing proteins might dictate the distinct physiological effects induced by the two βAR subtypes. 

Each of the four PDZ domain containing proteins above described, although binding to the same sequence of the β_1_AR, exerted different effects both on receptor trafficking and signaling. It is noteworthy that these four proteins do not share the same tissue distribution, PSD-95 and MAGI-2 being almost exclusively found in the brain, whereas GIPC and CNasGEF being predominantly expressed in the heart. A recent proteomic screen aimed at providing an exhaustive view of PDZ-mediated interactions at the β_1_AR, confirmed the association with PSD-95, MAGI-2, GIPC and CAL, and identified two novel ones, SAP97 and MAGI-3 [25]. MAGI-3 co-expression profoundly impaired β_1_AR-mediated ERK1/2 activation. 

To assess the GPCR selectivity of these interacting proteins a recent study used a library of 59 GPCR C-tails, among which those of the β_1_ and β_2_AR, in a pull-down assay [26]. This approach identified the lysosomal targeting protein GASP1 (G protein-coupled receptor-Associated Protein) as a potential interacting partner of the β_1_AR, but this finding was not explored further. GASP1 had been previously identified in a yeast two-hybrid screen as a protein interacting with the C-tail of the δ opioid receptor and shown to interfere with the post-endocytic sorting of the receptor. It was later discovered that GASP1 is member of a family of ten proteins displaying some sequence similarities whose functional implications are still largely unknown (reviewed in [27]). Whereas GASP1 can interact with several GPCRs other proteins of this family have been shown to modulate transcription. Considering these two important features, the GASP proteins might represent a promising area of investigation in the GPCR field. 

Another protein recently shown to interact with the β_1_AR and influence it’s trafficking is golgin-160 which is a ubiquitously expressed Golgi membrane protein [28]. Golgin-160, whose function in Golgi structure is unknown, can interact with the third intracellular loop of the β_1_AR and its depletion in cells leads to a significantly reduced cell surface expression of the receptor. 

An interesting interaction was recently found between the β_1_AR and the adaptor protein 14-3-3epsilon both in recombinant cells and heart [29]. The receptor/14-3-3 complex can compete with the interaction occurring between 14-3-3epsilon and the voltage-gated potassium channel, Kv11.1, and this might represent a mechanism involved in adrenergic regulation of cardiac repolarization. Whereas the wild type β_1_AR inhibits potassium current, a receptor mutant lacking its interaction with 14-3-3 can activate it. This is a fascinating finding highlighting the role of multiple protein interactions in fine tuning of the physiological effects mediated by the adrenergic receptors on cardiac rhythms. 

Another finding highlighting the potential functional impact of different signaling complexes in heart cells concerns the interaction of the β_1_ and β_2_AR subtypes with phosphodiesterases (PDE) [30]. Whereas the β_2_AR forms a complex with βarrestin and the PDE4D5 isoform, the β_1_AR can directly interact with the PDE4D8 isoform and the agonist can dissociate this complex. Both the receptor and PDE4 are regulated in a complex manner by the cAMP/PKA cascade and binding of PDE4D to the β_1_AR might allow for the control of cAMP levels in proximity of the receptor.

## PROTEINS INTERACTING AT THE β_2_AR 

PDZ domain-mediated interactions are also known to occur at the C-tail of the β_2_AR, which includes the type I PDZ-binding C-terminal sequence D-S-L-L. The first protein found to interact specifically with this sequence of the β_2_AR, but not with β_1_AR, was NHERF1 (Na+/H+ Exchanger Regulatory Factor 1), also named EBP50 (ERM- Binding Protein 50) [31]. This protein contains two PDZ domains and regulates the activity of the Na^+^/H^+^ exchanger type 3 (NHE3). In addition, NHERF factors represent a well established link between ERM (Ezrin-Radixin-Moesin) proteins and specific polytopic membrane proteins [32]. The β_2_AR/NHERF interaction might play a role in the receptor-mediated regulation of cellular pH. Typically, an increase of intracellular cAMP levels inhibits NHE3 activity (*via* PKA-mediated phosphorylation of NHERF), but the stimulation of the β_2_AR potentiates it. This is probably due to the fact that binding of the β_2_AR to NHERF relieves the NHERF-mediated inhibition of NHE3. 

NHERF can also be a link between the β_2_AR and other proteins which interact with NHERF, such as the platelet-derived growth factor (PDGF) receptor [33] and cystic fibrosis transmembrane conductance regulator (CFTR) [34]. Whereas PDGF receptor-mediated signaling is potentiated by its interaction with NHERF, the formation of the β_2_AR/NHERF complex might regulate PDGF receptor function. A macromolecular complex composed of the β_2_AR, CFTR and NHERF was shown to form in human airway epithelia and this complex might represent an important mechanism underlying the β_2_AR-stimulated increase of CFTR activity.

In addition to mediating these functional effects of the β_2_AR, the interaction with NHERF seems to play a direct role in receptor trafficking. The β_2_AR is a member of class A GPCRs, being characterized by rapid agonist-induced endocytosis and recycling back to the plasma membrane. It was demonstrated that disruption of either NHERF binding to the β_2_AR or NHERF binding to the actin cytoskeleton (mediated by NHERF/ERM interaction) caused missorting of endocytosed β_2_AR to the degradation pathway and prevented its recycling [35]. Actin depolymerization had a similar effect, implying that anchoring of the receptor to the actin cytoskeleton through NHERF and ERM proteins ensures its proper trafficking following endocytosis. A similar role for NHERF in directing the recycling of several other GPCRs was subsequently documented [32]. 

Another protein that associates with the distal part of the β_2_AR C-tail is NSF (N-ethylmaleimide-Sensitive Factor) [36]. Despite lacking PDZ domains, NSF also recognizes the extreme C-terminal sequence D-S-L-L and its association with the receptor is increased upon agonist treatment. NSF binding to the β_2_AR was shown to be required for both receptor internalization and recycling, but it is unclear whether a competition between NSF and NHERF binding to the receptor takes place and how this process is regulated.

The role of the PDZ binding sequence in the β_2_AR was validated in cardiac myocytes by the same group which investigated the β_1_AR-mediated effects on contraction rate [37]. Deletion of the PDZ binding motif in the β_2_AR abolished its coupling to Gi resulting in higher contraction rate, in contrast to the effect observed for the β_1_AR, where a similar mutation promoted Gi coupling and decreased contraction [24]. In agreement with previous findings, the mutated β_2_AR lacking interaction with NHERF was unable to recycle back to the plasma membrane. The experimental design did not allow to discriminate between NSF- and NHERF1-mediated effects. 

Stimulation of the β_2_AR raises intracellular cAMP levels leading to activation of protein kinase-A (PKA). The functional connection between the β_2_AR and PKA prompted the investigation on potential interactions between this receptor and PKA-anchoring proteins, or AKAPs. The first AKAP found to bind the β_2_AR was AKAP250, also known as gravin. Association of gravin to the receptor was increased upon agonist treatment [38] and involved the C-terminal portion of the receptor [39]. Suppression of gravin expression using an antisense strategy disrupted receptor resensitization and impaired its association to GRK2, βarrestin and clathrin, suggesting that gravin might serve as a scaffold bringing together kinases, phosphatases and proteins of the endocytic machinery. 

Another AKAP, AKAP79, directly and constitutively interacts with the β_2_AR and promotes its phosphorylation by PKA, which is obligatory for MAPK activation by the receptor [40]. The anchored PKA was further shown to phosphorylate GRK2 enabling its translocation to the membrane and subsequent phosphorylation of the β_2_AR [41]. Thus, AKAP79 is involved in the process of β_2_AR desensitization and internalization through the clathrin-dependent pathway. The molecular determinants of the receptor interaction with AKAPs were unfortunately not finely mapped and the question of the specificity of this interaction among adrenergic receptor subtypes has not been addressed yet.

An interaction of the β_2_AR with the adaptor protein Grb2 has also been reported [42]. This interaction was not identified through unbiased screening, but specifically tested based on the observation that insulin abolishes catechol-amine response by stimulating tyrosine phosphorylation of the β_2_AR. This phosphorylation creates an SH2 site on the β_2_AR which induces Grb2 binding to the receptor at Tyr250/254 thus leading to receptor-G protein uncoupling. Grb2 binding to the receptor was increased upon cell treatment with insulin and promoted also receptor internalization [43].

Interesting interactions have been found between the β_2_AR and ion channels involved in the regulation of membrane excitability. The β_2_AR was found to directly interact with the voltage-gated calcium channel Cav1.2 in hippocampal neurons [44]. A complex containing the β_2_AR, the Cav1.2, G protein, an adenylate cyclase, PKA and the PP2A phosphatase might represent a mechanism ensuring a specific and highly localized signaling process. 

It is well known that β_2_AR and PKA activation can increase BKCa activity in neurons, smooth muscle cells and other excitable cells. Recent findings indicate that the β_2_AR can interact with calcium sensitive K^+^ channels (BKCa) in a complex containing the receptor, BKCa and AKAP79 [45]. Thus, the β_2_AR might interact with both the Cav1.2 calcium channel and the BKCa calcium sensitive K^+^ channel, and these interactions might enable a highly localized control of membrane excitability.

A recent study reports a fascinating finding concerning the interaction between the β_2_AR and two proteins, the von Hippel-Lindau tumor suppressor protein (pVHL)-E3 ligase complex and the dioxygenase EGLN3 [46]. It is known that in response to hypoxia there is a decreased expression of β_1_AR in heart attributed to high levels of catecholamines whereas the β_2_AR abundance increases suggesting a direct regulation of this receptor by oxygen. The metallo-sensory enzyme dioxygenase EGLN3 transduces O_2_-responsive signals through modifications of target proteins. Thus, the interaction of EGLN3 with the β_2_AR results in hydroxylation of Pro382 and Pro395 of the receptor. After hydroxylation of the β_2_AR, pVHL-E3 ligase is recruited triggering ubiquitiny-lation of the β_2_AR and its degradation. This finding highlights the broadness and complexity of protein-protein interactions that can be explored to understand receptor function and regulation.

## PROTEINS INTERACTING AT THE β_3_AR

The β_3_AR subtype was cloned several years after the other βAR subtypes and its distribution seems to be restricted to adipose tissue, skeletal and smooth muscles. For these reasons little is known to date about the specific interactions of this AR subtype. One cytoplasmic partner has been identified, namely the tyrosine kinase Src, that can bind through its SH3 domain to the polyproline region in the third intracellular loop and the C-tail of the receptor [13]. The direct binding of Src of the β_3_AR seems required for ERK1/2 activation by the receptor. Previous reports from the same group had demonstrated an involvement of Src in MAPK activation by the β_2_AR, but this effect was mediated by the interaction of Src with βarrestin. As mentioned above, direct Src binding to the β_3_AR occurred through its SH2 domain on the phosphotyrosine Tyr350. The paucity of data regarding β_3_AR interactions leaves open an entire field of investigation in the signaling of this receptor.

## PROTEINS INTERACTING AT THE α_1A_AR

Few interactions have been shown to occur selectively at the α_1a_AR subtype. Yet, this receptor contains a PDZ binding sequence G-E-E-V at its C-terminus that can be expected to give rise to PDZ-domain mediated interactions. An early report, at the issue of a yeast two-hybrid screen, identified the type III PDZ domain of nNOS (neuronal nitric oxide synthase) as a potential α_1a_AR interacting protein [47]. However, co-immunoprecipitation studies, while confirming this interaction, failed to highlight selectivity for the α_1a_AR subtype since all three α_1_AR subtypes could be co-immunoprecipitated with nNOS and this even when they were lacking their C-terminus. This interaction appeared to be without apparent physiological implications in spite of the known role of NO in the regulation of blood pressure and of nNOS as local metabolic inhibitor of α_1_AR-mediated vasoconstriction. 

Another study reported that mammalian tolloid (mTLD) could interact with the α_1a_AR [48]. The CUB5 domain of mTLD, a zinc-finger matrix metalloprotease of the astacin family, interacted with α_1a_AR C-tail in a yeast two hybrid screen. The interaction was specific for the α_1a_AR and the binding region on the receptor could be narrowed down to a sequence of 37 aminoacids. Overexpression of mTLD reduced the number of cell surface receptors without affecting total receptor level or affinity when transiently expressed in HEK293 cells. mTLD also appeared to facilitate calcium signalling evoked by phenylephrine. No mechanism was proposed to account for the observed phenomena.

Interesting prospects were opened by the report of the direct interaction between RGS2 (Regulator of G protein Signaling 2) and the third intracellular loop of the α_1a_AR [49]. RGS proteins are well characterized inhibitors of heterotrimeric G protein function, acting as GAPs (GTPase activating proteins) to increase the rate of GTP hydrolysis at Gα subunits and thus terminate signaling. More than 30 RGS proteins have been identified so far, but many RGS proteins can non-selectively bind to and inhibit Gαi/o and Gαq11 in reconstituted systems, suggesting that other factors may regulate their specificity for a particular signaling pathway. RGS2 was found to interact with the α_1a_AR third intracellular loop confirming what previously shown for other Gq-coupled receptors, namely the cholinergic muscarinic M1, M3 and M5 receptors [50] and it inhibited agonist-induced inositol phosphate responses without affecting ligand binding.

## PROTEINS INTERACTING AT THE α_1B_AR 

Two main interacting partners were pulled out of a yeast two-hybrid screen for the α_1b_AR: the μ2 (or AP50) subunit of the clathrin adaptor complex AP2 [51] and ezrin, a member of the ERM protein family [52]. The AP2 complex is part of the endocytic machinery mediating clathrin-dependent endocytosis of membrane proteins and it is recruited to agonist-activated GPCRs through the intermission of βarrestins. Interactions of the AP50 subunit are dependent upon a YxxF motif present in the cargo protein. However, in the case of the α_1b_AR, binding of AP50 relied on a basic stretch of eight arginines in the proximal C-tail of the recaptor. Direct association of the α_1b_AR to AP50 contributed to the agonist-induced internalization of the receptor as demonstrated by the fact that a receptor mutant lacking the AP50 binding motif was delayed in internalization. The presence of the eight arginine motif in the C-tail of a GPCR is not common, which rules out the hypothesis that direct AP50 interaction is a common mechanism for clathrin-mediated endocytosis. Interestingly, this feature is shared by the α_1d_AR, which contains a stretch of seven positive charges in its C-tail, but no studies were undertaken using this receptor subytpe.

In addition to AP50, the same yeast two-hybrid screen identified ezrin as a potentially direct binding partner of the α_1b_AR [52]. Ezrin belongs to the ERM family of proteins, primarily described as linkers between membrane proteins and cortical actin. Ezrin was also shown to be involved in the remodelling of the actin cytoskeleton, in the modulation of Rho signaling (by binding to Rho GTP dissociation inhibitor (GDI) and through direct association to several Rho GTP/GDP exchange factors (GEFs) as well as in anchoring of PKA. Ezrin interactions with polytopic membrane proteins generally occur through the adaptor proteins EBP50 (NHERF1) and E3KARP (NHERF2), but direct contacts were also described between ezrin and proteins such as the Na+/H+ exchanger type 1 (NHE1) and podocalyxin. So far, a role for the ERM proteins in GPCR trafficking was inferred from the finding that NHERF1 binding to some GPCRs promoted their recycling, depending on its binding to ERM proteins. The α_1b_AR is the first GPCR for which a direct interaction with ezrin has been found. Disruption of this interaction by overexpression of a dominant negative mutant of ezrin inhibited receptor recycling after internalization, as did actin depolymerization. Thus, the involvement of ERM proteins in GPCR recycling, through either a direct or indirect interaction with the receptor, might represent a general paradigm for the regulation of GPCR trafficking. 

Intriguingly, ezrin and AP50 shared the same binding site on the α_1b_AR C-tail, consisting in the eight arginines stretch positioned after the putative palmitoylation site. In pulldown experiments the binding domain of ezrin and that of AP50 competes with each other for the same binding site of the receptor C-tail (unpublished data). How these events are regulated within the cell is a matter that awaits further inquiry.

Another protein, the receptor for globular "Heads" of c1q (gC1qR), was reported to interact with the same argininerich sequence in the α_1b_ and the α_1d_AR [53]. gC1qR is a glycoprotein mainly displaying intracellular localization, but also present on the surface of macrophages and T cells through anchoring to β-integrin, where it is part of a complement receptor. No functional relevance was demonstrated for its interaction with the α_1b_ or α_1d_AR.

## PROTEINS INTERACTING AT THE α_1D_AR

The α_1d_AR was for a long time a “poor relative” to the other α_1_AR subtypes, the α_1a_ and α_1b_ because poorly expressed at the cell surface in heterologous systems, probably because of its long N-terminus. This peculiarity hampered the investigation of its potential interactions with other proteins. Apart from the above mentioned interaction with gC1qR, whose functional implications are unknown [53], another interacting partner of the α_1d_AR was α-syntrophin [54]. α-syntrophin, a protein containing one PDZ domain and two PH (pleckstrin homology) domains, specifically recognized the C-tail of the α_1d_AR, but not that of the α_1a_ or α_1b_, in the yeast two-hybrid assay. The PDZ domains of syntrophin isoforms α, β1 and β2, but not γ1 or γ2, could interact with the α_1d_AR C-tail. The α_1d_AR possesses the C-terminal sequence E-T-D-I, whose mutation impaired syntrophin binding to the receptor and markedly decreased norepinephrine-induced inositol phosphate accumulation. This mutation also dramatically decreased receptor expression levels. Interestingly, syntrophins seemed to interact equally well with intracellular or surface-expressed α_1d_AR receptors. Taken altogether these results suggested that syntrophins act to maintain the stability of the α_1d_AR through a PDZ-mediated interaction.

## PROTEINS INTERACTING AT THE α_2_AR SUB-TYPES

The three α_2_AR receptor subtypes (α_2_A α_2_B and α_2_C) are coupled to the Gi/o family of heterotrimeric G proteins, and hence to the inhibition of adenylyl cyclase and voltage sensitive calcium channels, and to the activation of potassium channels. They are differently expressed in various tissues including the basolateral membrane of polarized renal epithelial cells where differences in the targeting and trafficking of the three subtypes have been found. The proper basolateral retention of the α_2_AR receptor subtypes is dependent upon the integrity of their third intracellular loop, a finding which prompted the search for partners interacting with this region of the receptors. A gel overlay strategy using *in vitro* translated i3 loops of the α_2_AR receptors highlighted the interaction of the zeta isoform of 14-3-3-proteins [55]. 14-3-3 proteins are ubiquitous and involved in the regulation of a number of signaling pathways, among which the Ras/ MAPK cascade. 14-3-3 interacted preferentially with the α_2A_ and α_2B _than with the α_2C_. A detailed study of the sequence requirements for this interaction at the α_2A_AR did not identify a single linear motif suggesting that a three dimensional structure in the i3 loop is needed for 14-3-3 binding [56].

An important binding partner of all three α_2_AR subtypes is spinophilin [57, 58]. This interaction was specifically tested with the rationale that spinophilin is enriched beneath the basolateral membrane of MDCK cells. Spinophilin binding to α_2A_AR was enhanced by agonist treatment and the region responsible for binding was loosely mapped to the N- and C-terminal ends of the i3 loop [56]. Interaction with spinophilin contributed to the cell surface stabilization of the α_2B_AR subtype, a receptor that displays the unique property of being first randomly delivered to both apical and basolateral side of the cell, with a much faster apical *versus* basolateral turnover which ends up in its accumulation on the basolateral side. Apical delivery of a spinophilin subdomain extended the half-life of the α_2B_AR in this region; further-more, agonist-stimulated internalization of the receptor was accelerated in fibroblats derived from spinophilin knock-out mice [59]. Presumably, similar effects could occur at the other two α_2_AR subtypes. An explanation of this effect came from the finding that spinophilin blocks GRK2 association to the α_2B_ARs thus inhibiting receptor endocytosis [60]. 

Spinophilin was also found to interact with other GPCRs, including the α_1b_AR, as well as with the N-terminal domain of RGS proteins (RGS1, 2, 4 and 16) which participates in GPCR recognition [60, 61]. Thus spinophilin might represent an interesting functional bridge between RGS and α_1_AR subtypes that don't bind RGS, like the α_1b_AR. In fact, it has been found that spinophilin increases the RGS2-induced inhibition of the α_1b_AR calcium response. In support of this finding, a constitutively active α_1b_AR mutant did not bind spinophilin and was resistant to inhibition by RGS2. Similar resistance to RGS2 inhibition was found to occur in spinophilin knock-out cells. These data offer a glimpse into a potentially more general regulatory mechanisms of GPCR function by spinophilin.

The most recent protein found to interact with α_2_ARs is ubiquitin carboxyl-terminal hydrolase-L1 (Uch-L1), a protein associated with Parkinson disease [62]. Uch-L1 binds preferentially to the α_2A_AR subtype and its overexpression inhibits the receptor-induced activation of MAPK. This interaction might have implications in the neuroprotective effect of α_2_ARs which needs to be further investigated.

## OLIGOMERIZATION OF THE ADRENERGIC RECEPTORS

Findings in the last decade challenged the widely held view of GPCRs functioning as monomeric units [7]. Early biochemical studies showing higher molecular weight recaptor bands migrating in SDS-PAGE gels, stabilized by cross-linking, suggested that adrenergic receptors might form oligomers. Afterwards, co-immunoprecipitation of differentially tagged GPCRs or functional complementation of pairs of co-expressed inactive receptor mutants provided stronger evidence in favor of GPCR oligomers. The widespread use of biophysical techniques such as FRET or BRET between GPCRs carrying the appropriate pair of fluorescent/ bioluminescent labels suggested oligomerization of a variety of GPCRs. Each technique employed has its own shortcomings: whereas co-immunoprecipitation cannot rule out indirect interactions, energy transfer techniques can only certify that the two partners are in close proximity, not necessarily in immediate contact. Therefore, whether receptor oligomerization involves direct interactions among receptor monomers *versus *their increased proximity in micro-domains of the cell membrane cannot be unequivocally demonstrated. Despite the fact that the precise molecular events are not fully understood, the term of GPCR oligomerization is largely accepted to indicate the existence within the cell membrane of macromolecular complexes formed by two or more receptor monomers. It is important to highlight that most studies have been performed in recombinant cells overexpressing the receptors with very few examples of oligomerization occurring in physiological systems.

### Homo-Oligomerization

Within the adrenergic receptor family, the β_2_AR was the first member for which homo-oligomerization was described, using co-immunoprecipitation of epitope-tagged receptors [63]. BRET technology was then adapted for the purpose of showing the existence of receptor oligomers at the cell surface and of quantitatively assessing the extent of oligomerization [63, 64]. Homo-oligomerization was therefore demonstrated for the β_2_AR [63, 64], β_1_AR [65], α_1a_ and α_1b_ [66, 67], α_1d_ [67], α_2a_ and α_2c _[68], and for the α_2b_ [69] AR subtypes.

Whereas the stoichiometry of these complexes is hard to assess with either biochemical or biophysical methods used so far, the structural architecture of receptor oligomers has been addressed by some studies using computational, pharmacological and biochemical approaches. Two types of receptor-receptor interaction have been proposed, involving either lateral contact or domain swapping. A model was proposed for a β_2_AR dimer where the two receptor monomers swapped helices V and VI [70]. Other studies pointed at helices I and VII [66] or I/II and III/IV [71] as the probable interface for receptor-receptor contact in the case of the α_1b_ AR. Neither the C-terminal tail of the α_1b_AR nor its glycosylation state or the presence or absence of a glycolphorin dimerization motif GxxxG in the transmembrane domains affected its oligomerization [66]. This was in contrast with the previous report of Hébert *et al.*, showing that helix VI containing the GxxxGxxxG motif was responsible for oligomerization of the β_2_AR [63].

The functional implications of AR homo-ligomerization have been explored by several studies. Preventing receptor association has been technically difficult with few exceptions. In the case of the β_2_AR, a peptide derived from helix VI was shown to disrupt dimerization and its use was demonstrated to impair isoproterenol-stimulated cAMP production by the β_2_ AR [63] implying that β_2_oligomers would be the functional form of the receptor.

Among the functional implications of AR homo-oligomers, different studies investigated their localization within the cell. BRET studies suggested that β_2_AR oligomers are present at the plasma membrane [64]. Biotinylation with a membrane-impermeable showed expression of α_1a_, α_1b_ and α_1d _homodimers at the cell surface [67]. Moreover, homo-oligomerization of the β_2_AR seems to be a prerequisite for its plasma membrane targeting [72]. In fact, the peptide derived from helix VI of the β_2_AR, which blocks receptor oligomerization, prevented normal targeting of the receptor to the plasma membrane. These results imply that receptor oligomers form as early as the stage of their synthesis in the endoplsmic reticulum and that oligomerization influences their maturation and trafficking ability from then onwards. Indeed, co-expression with a recycling-defective mutant diverted wild type β_2_AR from normal recycling to the plasma membrane to the proteolytic degradative pathway [73]. Similar data were obtained for the α_2B_AR subtype; when co-expressed with a mutant deficient in cell surface targeting, the wild type α_2B_AR remained trapped in the endoplsmic reticulum [69].

A matter of debate has been the regulation of the oligomeric status of GPCRs by ligands. One among the first reports indicated that agonist binding could increase the amount of β_2_AR dimers [63], corroborated by BRET data [64]. In contrast, agonist treatment had no apparent effect on the oligomerization of α_1_AR subtypes [66, 67]. Furthermore, constitutively active or non-functional α_1b_AR mutants displayed the same propensity to oligomerize as the wild type receptor [66], indicating that the activation state of the receptor is irrelevant for this process.

### Hetero-Oligomerization

Different AR subtypes are often co-expressed in the same cells and cross-talk among them can occur. Various studies addressed the hypothesis that hetero-oligomerization could account for cross-talk effects. A number of interactions between different AR subtypes or between ARs and more distantly related GPCRs have been reported (Table **2**). 

The adrenergic receptor subtypes seem to display selectivity of interaction and hetero-oligomers do not form for any two receptor combination. Within the AR family, the following main hetero-oligomers have been found: β_1_/β_2_ [74], β_2_/β_3_ [75], α_1b_/α_1a_ and α_1b_/α_1d_, (but not α_1a_/α_1d_) [66, 67, 76], α_1b_/β_2_ [66], α_1d_/β_2_ [77], α_2A_/α_2C_ [68], α_2A_/β_1_ [78] and α_2C_/β_2_ [79]. 

In addition, the following main combinations have been described between AR subtypes and other GPCRs: β_2_AR/ olfactory receptor [80], β_2_AR/δ opioid and β_2_AR/κ opioid [81], βAR/AT1 angiotensin II [82], β_2_AR/5-HT4 serotonin [83], β_2_AR/EP1 prostaglandin [84], β_2_AR/CB1 cannabinoid [85], α_2A_/μ opioid [86] and α_2A_/δ opioid [87]. Very recently a macromolecular complex including the β_2_AR, Gs, PKA, adenylate cyclase and the AMPA glutamate receptor, mGluR1, has been described [88].

Hetero-oligomerization was found to have various functional effects upon the behavior of individual receptors, ranging from regulated targeting to modified pharmacological, signaling or trafficking profile in recombinant systems. Co-expression of the α_1d_AR with the α_1b_AR [76] or the β_2_AR [77] was able to rescue surface expression of the α_1d_AR, the majority of which is intracellular when expressed alone in various cell lines. A similar phenomenon was already observed for the GABA-B receptor and for the calcitonin/adrenomedullin receptor, both receptors needing hetero-dimerization in order to be properly targeted to the plasma membrane. Interestingly, the interaction with the α_1b_AR modified the pharmacological profile of the α_1d_AR which looses its affinity for its selective ligand BMY7378 when it is co-expressed with the α_1b_AR. The α_1b_/α_1d_ dimer behaves as a single functional entity with increased response to norepinephrine relative to either monomer alone. The α_1d_AR receptor was long supposed to be little expressed in the heart, as its selective ligand BMY7378 could detect only minimal levels of the receptor. However, these findings should be considered in a new light, given that the α_1b_ and α_1d_AR subtypes co-exist in this tissue and the pharmacological profile of the α_1d_AR might be different than expected because of oligomerization. 

Hetero-dimerization of the β_2_AR with the α_2C_AR, which is normally poorly expressed in recombinant cells, increased the expression at the cell surface and ERK1/2 signaling of the α_2C_AR [79]. Coexpression of the α_2C_AR with more than twenty-five GPCRs revealed that only the β_2_AR induced this effect. Coexpression of the β_2_AR with the olfactory receptor (M71) also promoted the cell surface localization of this receptor which is often co-expressed with the β_2_AR in olfactory neurons [80]. Again, only the β_2_AR among the AR subtypes had this effect. 

Beyond its effect on receptor targeting to the cell surface, hetero-oligomerization seems to have an impact on various aspects of receptor trafficking and signaling. For example, several studies have shown that receptor monomers can mutually influence their endocytosis pattern. Whereas the α_1b_AR undergoes agonist-induced internalization, the α_1a_AR does not. However, when the two AR subtypes were co-expressed forming heterodimers, the endocytosis of each monomer could be triggered by stimulation of the other [66]. Colocalization of the two monomers could be seen in endocytic vesicles suggesting that the α_1a_/α_1b_ dimers remained stable throughout the endocytosis process. 

Strikingly, in β_1_/β_2 _heterodimers, the internalization of the β_2_AR within the complex was inhibited. Furthermore, β_2_AR-induced ERK activation was equally blocked by co-expression of β_1_AR [78]. Similar results were also found for the β_2_/β_3_ heterodimer [79] in which β_2_AR internalization was impaired. The β_3_AR is resistant to agonist-promoted endocytosis and, like the β_1_AR, acted as a dominant negative on β_2_AR internalization.

In α_2A_/β_1_ heterodimers, stimulation of the α_2A_AR triggered the internalization of the β_1_AR [78]. In addition, the β_1_AR within the heterodimer displayed altered pharmacology. In α_2A_/α_2C _heterodimers, the GRK-dependent phosphorylation and βarrestin recruitment at the α_2A_AR, were inhibited [68].

Functional cross-talk was also described between the β_2_AR and the opioid receptors (OR) that are coupled to stimulatory and inhibitory G proteins, respectively. Whereas in the β_2_/δ opioid dimer the β_2_AR and δOR could facilitate the endocytosis of each other, in the β_2_/κ opioid dimer the internalization of the β_2_AR was inhibited. Moreover, isoproterenol-induced MAPK activation was diminished in presence of κOR, showing that the κOR acts as a dominant negative modulator of the β_2_AR [81]. 

The same group explored the possible interaction between α_2_AR and opioid receptors that colocalize in neurons and affect the nociceptive response. The α_2A_/μ opioid could be isolated from recombinant cells as well as from primary neurons. In the α_2A_/μ opioid dimer the activation of each monomer increases G protein and MAPK signaling whereas the activation of both monomers decreases it [86]. In addition, when the receptors were expressed in a neuronal cell line the α_2A_AR increased the δOR-mediated neurite outgrowth [87]. These results support the notion that α_2_AR and opioid receptors are synergic in spinal analgesia as also demonstrated by the decrease in morphine-induced analgesia in α_2A_-/- knock out mice. Thus, the physical association between the α_2_AR and opioid receptors might explain their functional interaction. 

Some interesting findings have been reported concerning the potential implications of βAR hetero-oligomerization with other GPCRs in native tissues. Whether in native tissues simultaneous synthesis of hetero-dimer partners can occur or different receptors are simply clustered in the same cell membrane compartment is not known.

In freshly isolated mouse cardiomyocytes, interesting effects were reported on contractility induced by angiotensin II (AgII) acting at AT1 receptor or isoproterenol acting at βAR [82]. Whereas the beta-blocker propranolol could inhibit the effect of AgII, the AT1 blocker valsartan inhibited that of isoproterenol. This transinhibitory effect of the two antagonists seemed related to inhibition of receptor coupling to its cognate G protein. Biochemical experiments on β_2_AR and AT1 receptor expressed in HEK cells indicated that the two receptors could be co-immunoprecipitated thus suggesting that hetero-oligomerization of the two receptors could be the basis of their functional interaction in heart.

Hetero-oligomerization between the β_2_AR (coupled to Gs) and the EP1 prostaglandin receptor (coupled to Gq) has been observed in airway smooth muscle [84]. In mouse tracheal rings, activation of the β_2_AR induces muscle relaxation whereas stimulation of EP1 alone has no effect on contraction. However, stimulation of the EP1 receptor profoundly reduced the β_2_AR-induced muscle relaxation and cAMP accumulation. The modulatory effect of EP1 receptor on the β_2_AR might depend on the interaction between the two receptors which form heterodimers in airway smooth muscle cells.

Hetero-oligomerization has also been recently observed using BRET between the β_2_AR and the CB1 cannabinoid receptor expressed in recombinant cells [85]. The formation of oligomers could be increased by a CB1 inverse agonist. Interestingly, co-expression of the two receptors resulted in increased localization at the cell surface and decreased constitutive activity of the CB1 receptor. Complex functional interactions on signaling between the two receptors have been found both in the recombinant system and in primary human trabecular meshwork (HTM) cells from the eye, a tissue in which the two receptors are natively co-expressed. Beyond mutual effects on ERK activation, activation of one receptor induces cross-desensitization of the other both in recombinant and HTM cells. This might be relevant in drug therapy since CB1 agonists and β_2_AR antagonists can both reduce intraocular pressure. 

Very recently, an elegant study demonstrated that the β_2_AR can form a signaling complex with the GluR1 subunit of the AMPA glutamate receptor including also the trimeric Gs protein, adenylate cyclase and protein kinase A [88]. This complex seems to be important to allow β_2_AR-induced phosphorylation of the GluR1 which results in increased GluR1 cell surface expression and current amplitudes.

## CONCLUSIONS AND PERSPECTIVES

The AR subtypes have been found to interact with several proteins (Table **1**) as well as to form homo and hetero-oligomers (Table **2**). A critical approach in analyzing all these interactions should address a number of questions: is the interaction selective for one receptor or common to others? is it occurring in specific tissues? what are its functional implications? Addressing these questions is important to assess whether the interface of a receptor with a specific protein could be an interesting target of pharmacological intervention. For most interactions described at the AR subtypes, the answers to these questions are far from being answered.

Most of the interactions involving the AR subtypes have been found using various screening approaches (proteomic, yeast two hybrid or pull-down experiments) without a specific bias towards the interactions searched. In most studies, the investigation of the interactions has been performed in recombinant systems in which only a limited number of functional parameters common to all GPCRs can be explored, i.e. trafficking, signaling through known pathways, receptor pharmacology. This resulted in the identification of a number of proteins affecting receptor endocytosis (endophilins, PSD-95, MAGI-2, NSF, AKAP250, Grb2, AP50) and few others stabilizing receptor expression at the cell surface (golgin-160, spinophilin, syntrophin), favoring recycling (ezrin, NHERF) or regulating receptor coupling to G proteins (RGS2) (Table **1**). Most of these interactions have been investigated only at a specific AR subtype with few exceptions. For example, the role of spinophilin in stabilizing receptor expression at the cell surface seems a property of all three α_2_AR subtypes (α_2A_, α_2B_ and α_2C_) [55, 56]. Spinophilin has been found to interact also with the α_1b_AR displaying, however, a different effect, i.e. the modulation of calcium signaling. Spinophilin certainly represents a protein of pharmacological interest which should be further investigated because of its well established role in targeting the α_2_AR subtypes in polarized cells as well as in regulating their function. 

It seems quite evident that proteins containing the PDZ domain can interact with both the β_1_ and β_2_AR. However, some PDZ domain containing proteins seem to display a selectivity towards either one of two receptors. For example, NHERF interacts with the C-tail of the β_2_AR, but not with the β_1_[31]. PSD-95 interacts selectively with the β_1_AR being both proteins expressed in post-synaptic densities [18]. The interaction of PDZ proteins with the β_1_ and β_2_AR is also one of the few examples in which the functional implication has been investigated in a physiological system, i.e. mouse cardiac myocytes [24, 37]. Deletion of the PDZ binding motif in the β_2_AR abolished its coupling to Gi resulting in higher contraction rate, in contrast to the effect observed for the β_1_AR, where a similar mutation promoted Gi coupling and decreased contraction. Because of the functional relevance of these interactions, PDZ domain containing proteins might represent interesting targets to pharmacologically interfere with trafficking and signaling of either the β_1_ or β_2_AR. Recently, some success has been reported in designing small peptides blocking PDZ interactions [89]. In addition, despite the wide distribution of PDZ proteins, the overlapping expression of interacting partners might be restricted to some tissues and this could be functionally relevant. More detailed studies on this important family of proteins in the regulation of AR subtypes are required and might have important implications.

Beyond the various interacting proteins found using different screening approaches, few highly interesting interactions have been found in elegant studies exploring the activity of the β_2_AR in neuronal cells. The β_2_AR was found to directly interact with the voltage-gated calcium channel Cav1.2 in hippocampal neurons [44]. It was reported that the β_2_AR can also interact with calcium sensitive K^+^ channels (BKCa) in a complex containing the receptor, BKCa and AKAP79 [42]. Thus, the β_2_AR might interact with both the Cav1.2 calcium channel and the BKCa calcium sensitive K^+^ channel, and these interactions might enable a highly localized control of membrane excitability. Another recent study has reported that the β_2_AR can form a signaling complex with the GluR1 subunit of the AMPA glutamate receptor including also the trimeric Gs protein, adenylate cyclase and protein kinase A [88]. This complex might underlie the facilitating effect of βARs on long term potentiation mediated by AMPA receptors.

Altogether, these latter studies clearly indicate that the full elucidation of signaling events in time and space will depend on a much deeper understanding of the interactions among receptors and signaling molecules. Developing drugs acting at distinct receptor-protein interfaces might represent an approach to achieve more cell specific pharmacological effects. However, this field is still at an early stage because of the complexity of studying these events in physiological cell systems as demonstrated by the limited number of studies published so far. Therefore, the fine-tuning of GPCR activity by receptor-interacting proteins is a very promising area of investigation in which a lot remains to be explored [90]. Studies in recombinant systems can certainly provide some useful information, but the real challenge concerns the possibility of exploring the functional implications of a variety of interactions in different tissues and physiological conditions. Without these studies it will be difficult to assess which of these interactions are druggable.

## Figures and Tables

**Table 1.** Proteins Selectively Interacting with Distinct Adrenergic Receptor Subtypes

Receptor	Partner	Binding Site	Functional Role	Refs.
β_1_	endophilins	i3 loop (Pro-rich)	↑ endocytosis	[17]
β_1_	PSD-95	C-tail (ESKV)	↓ endocytosis; β_1_ /NMDA receptor association	[18]
β_1_	MAGI-2	C-tail (ESKV)	↑ endocytosis; association to β-catenin	[20]
β_1_	GIPC	C-tail (ESKV)	↓ ERK activation	[21]
β_1_	CAL	C-tail (ESKV)	↓ cell surface expression	[22]
β_1_	CNrasGEF	C-tail (ESKV)	Ras activation	[23]
β_1_	MAGI-3	C-tail (ESKV)	↓ ERK activation	[25]
β_1_	GASP	C-tail (ESKV)	unknown	[26]
β_1_	golgin-160	i3 loop	↑ cell surface expression	[28]
β_1_	14-3-3	phospho-sites	regulation of K^+^ current	[29]
β_1_	PDE4D8	unknown	regulation of cAMP levels	[30]
β_2_	NHERF (or EBP50)	C-tail (DSLL)	regulation of NHE3; regulation of PDGF and CFTR activity; receptor recycling	[31]
β_2_	NSF	C-tail (DSLL)	↑ endocytosis	[36]
β_2_	AKAP250 (gravin)	C-tail	↑ endocytosis and resensitization; ↑ association to GRK2 and barrestin	[38]
β_2_	AKAP79	unknown	↑ agonist-induced phosphorylation by GRK2	[40, 41]
β_2_	Grb2	Tyr350/354	↑ endocytosis stimulated by insulin	[42]
β_2_	Cav1.2	unknown	↑ Ca^++^ current	[44]
β_2_	BKCa	unknown	↑ K^+^ current	[45]
β_2_	pVHL EGLN3	Pro382/395	O2-induced ubiquitinylation	[46]
β_2_	Src	i3 loop and C-tail (Pro-rich)	↑ ERK activation	[13]
α_1a_ α_1b_ α_1d_	nNOS	unknown	unknown	[47]
α_1a_	tolloid	C-tail	↓ surface expression	[48]
α_1a_	RGS2	i3 loop (K219-S220-R238)	↓ Gq signaling	[49]
α_1b_	AP50	C-tail (8 Arg)	↑ endocytosis	[51]
α_1b_	ezrin	C-tail (8 Arg)	↑ recycling	[52]
α_1b_	spinophilin	i3 loop	↓ Ca2^+^ signaling induced by RGS2	[61]
α_1d_	syntrophins	C-term (ETDI)	stabilization of receptor at cell surface	[54]
α_1b_ α_1d_	gC1qR	C-tail (Arg)	unknown	[53]
α_2A_ α_2B_ α_2C_	14-3-3 z	i3 loop	unknown	[55]
α_2A_ α_2B_ α_2C_	spinophilin	i3 loop	stabilization of receptor at cell surface; ↓ arrestin action	[55, 56]
α_2A_	Uch-L1	i3 loop	↓ MAPK activation	[62]

**Table 2.** Hetero-Oligomerization of the Adrenergic Receptors

Receptors	Trafficking	Pharmacology	Signaling	Refs.
β_1_/β_2_	no β_2_ endocytosis		↓ β_2_ ERK activation	[74]
β_2_/b3	no β_2_ endocytosis		no Gi/o coupling	[75]
β_2_/Olf	↑ Olf surface expression; co-endocytosis			[80]
β_2_/δOR	co-endocytosis			[81]
β_2_/kOR	no β_2_ endocytosis		↓ β_2_ MAPK activation	[81]
β_2_/AT1	trans-inhibition of endocytosis by antagonists		trans-inhibition of G protein coupling by antagonists	[82]
β_2_/5-HT4				[83]
β_2_/EP1			↓ β_2_ smooth muscle relaxation	[84]
β_2_/CB1;	↓ constitutive endocytosis of CB1; co-endocytosis		mutual effects on signaling; cross-desensitization	[85]
α_1a_/α_1b_	co-endocytosis	no change		[66]
α_1b_/α_1d_	↑ α_1d_ surface expression	↓ α_1d_ affinity for selective ligands	↑ signaling	[67, 76]
α_1d_/β_2_	↑ α_1d_ surface expression; co-endocytosis			[77]
α_2A_/α_2C_			↓ α_2A_ phosphorylation & βarrestin recruitment	[68]
α_2A_/β_2_;_1_	co-endocytosis	altered β_1_ profile		[78]
α_2C_/β_2_	↑ α_2C_ surface expression		↑ α_2C_ ERK signaling	[79]
α_2A_/mOR			↑ signalling of each monomer	[86]
α_2A_/dOR			↑ δOR neurite outgrowth	[87]
